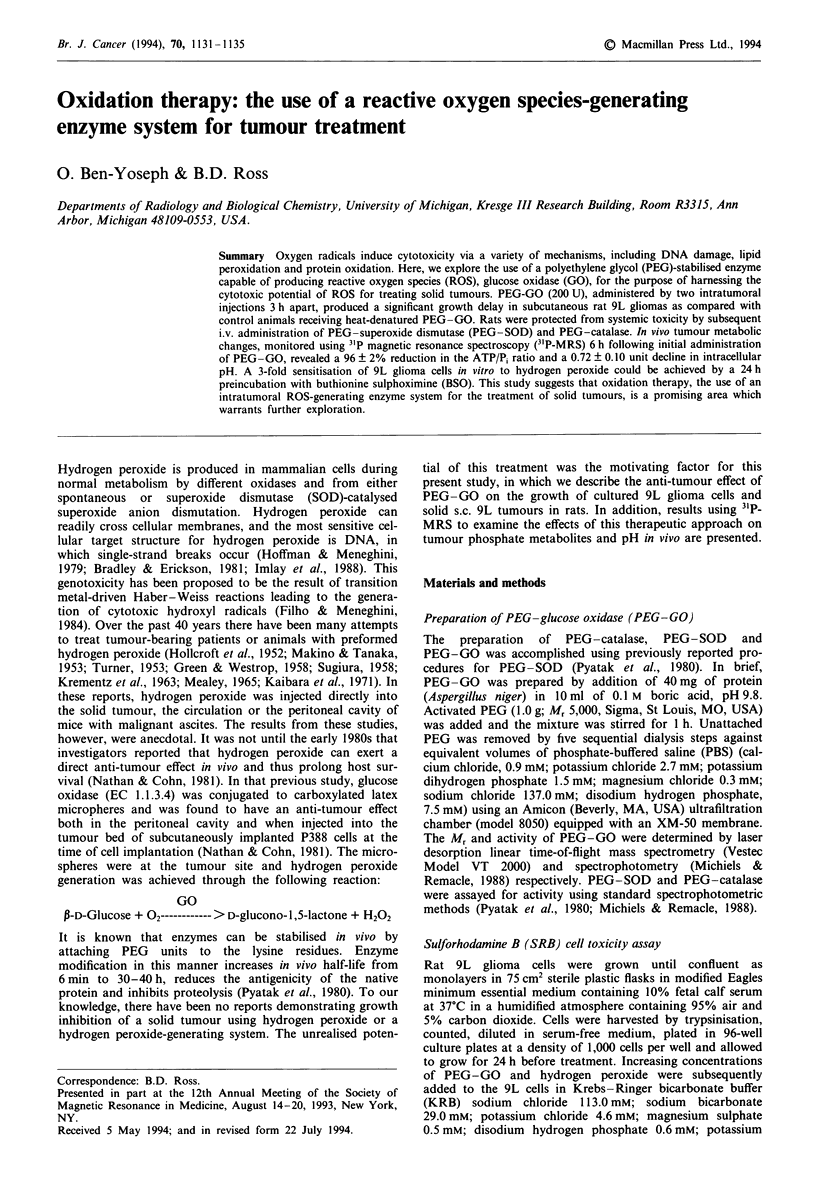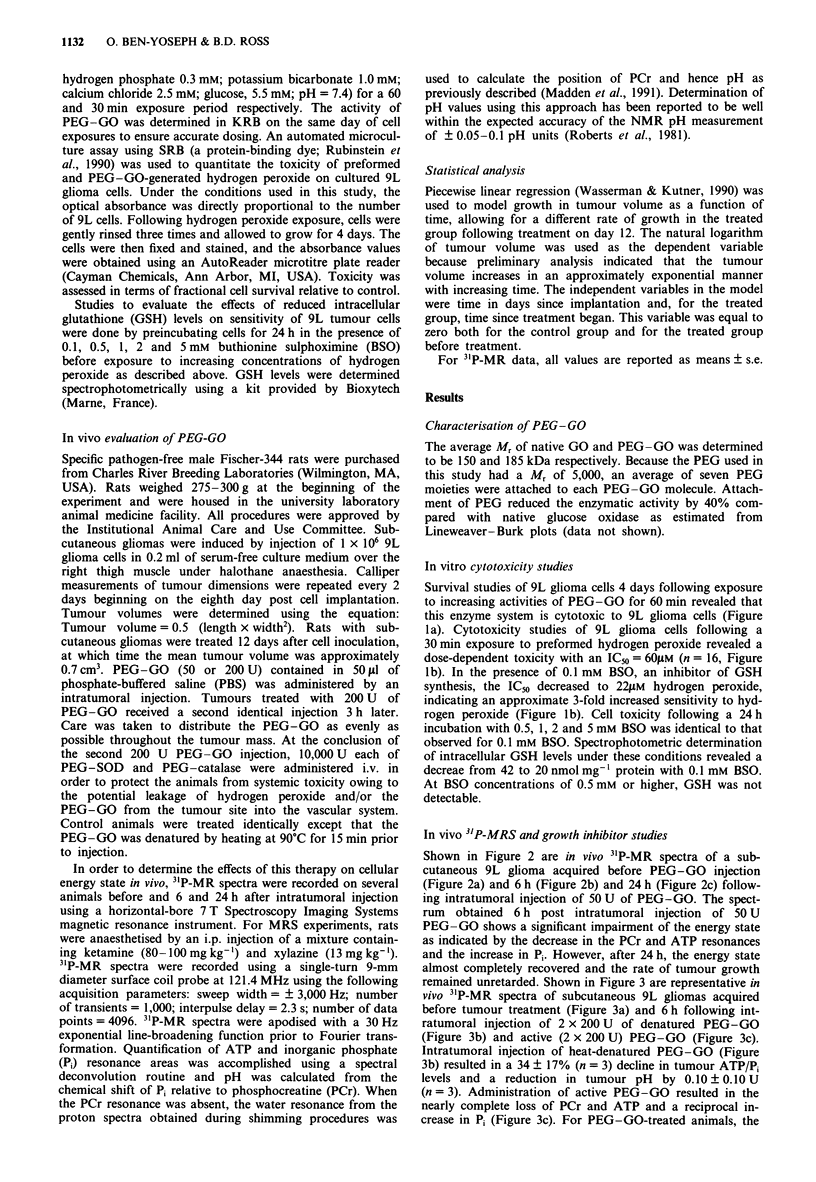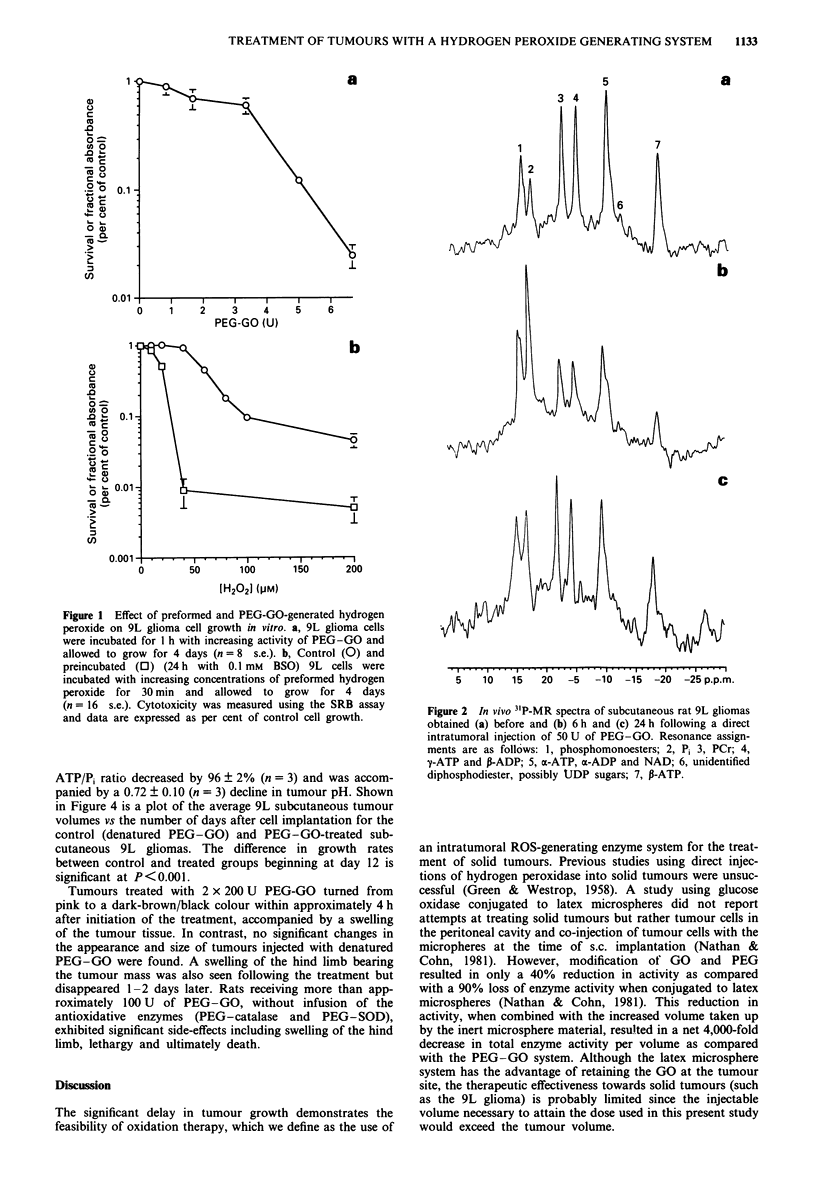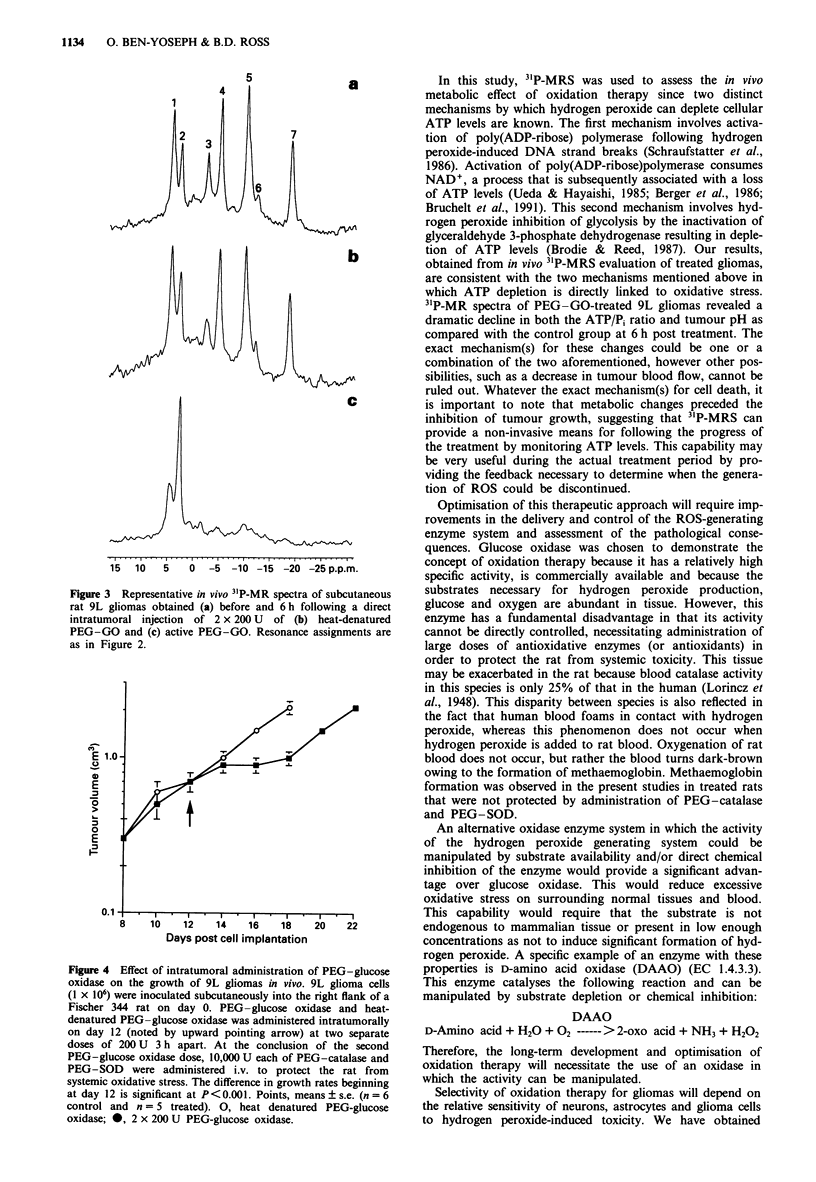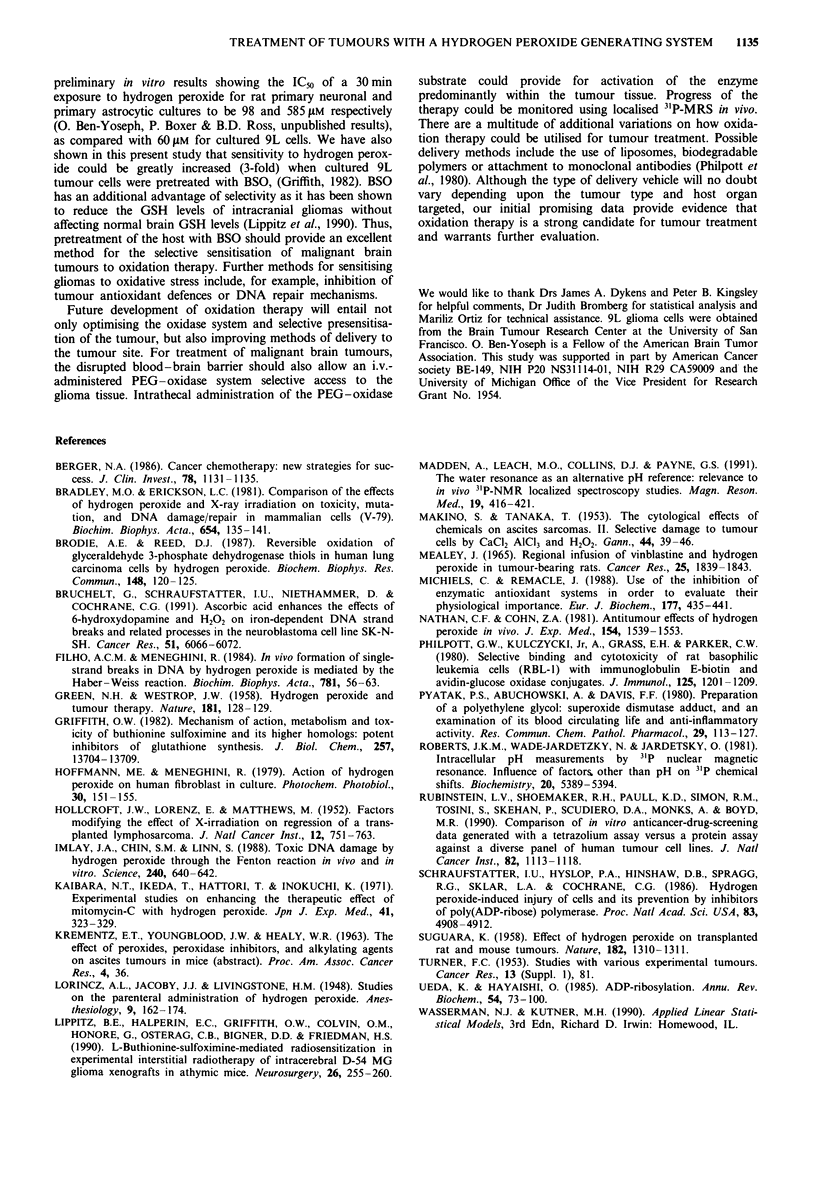# Oxidation therapy: the use of a reactive oxygen species-generating enzyme system for tumour treatment.

**DOI:** 10.1038/bjc.1994.460

**Published:** 1994-12

**Authors:** O. Ben-Yoseph, B. D. Ross

**Affiliations:** Department of Radiology, University of Michigan, Ann Arbor 48109-0553.

## Abstract

Oxygen radicals induce cytotoxicity via a variety of mechanisms, including DNA damage, lipid peroxidation and protein oxidation. Here, we explore the use of a polyethylene glycol (PEG)-stabilised enzyme capable of producing reactive oxygen species (ROS), glucose oxidase (GO), for the purpose of harnessing the cytotoxic potential of ROS for treating solid tumours. PEG-GO (200 U), administered by two intratumoral injections 3 h apart, produced a significant growth delay in subcutaneous rat 9L gliomas as compared with control animals receiving heat-denatured PEG-GO. Rats were protected from systemic toxicity by subsequent i.v. administration of PEG-superoxide dismutase (PEG-SOD) and PEG-catalase. In vivo tumour metabolic changes, monitored using 31P magnetic resonance spectroscopy (31P-MRS) 6 h following initial administration of PEG-GO, revealed a 96 +/- 2% reduction in the ATP/Pi ratio and a 0.72 +/- 0.10 unit decline in intracellular pH. A 3-fold sensitisation of 9L glioma cells in vitro to hydrogen peroxide could be achieved by a 24 h preincubation with buthionine sulphoximine (BSO). This study suggests that oxidation therapy, the use of an intratumoral ROS-generating enzyme system for the treatment of solid tumours, is a promising area which warrants further exploration.


*Br. J. Cancer* (1994), **70**, 1131–1135 © Macmillan Press Ltd., 1994

### Oxidation therapy: the use of a reactive oxygen species-generating enzyme system for tumour treatment

O. Ben-Yoseph & B.D. Ross

*Departments of Radiology and Biological Chemistry, University of Michigan, Kresge III Research Building, Room R3315, Ann Arbor, Michigan 48109-0553, USA.*

**Summary** Oxygen radicals induce cytotoxicity via a variety of mechanisms, including DNA damage, lipid peroxidation and protein oxidation. Here, we explore the use of a polyethylene glycol (PEG)-stabilised enzyme capable of producing reactive oxygen species (ROS), glucose oxidase (GO), for the purpose of harnessing the cytotoxic potential of ROS for treating solid tumours. PEG-GO (200 U), administered by two intratumoral injections 3 h apart, produced a significant growth delay in subcutaneous rat 9L gliomas as compared with control animals receiving heat-denatured PEG–GO. Rats were protected from systemic toxicity by subsequent i.v. administration of PEG–superoxide dismutase (PEG–SOD) and PEG–catalase. *In vivo* tumour metabolic changes, monitored using $^{31}P$ magnetic resonance spectroscopy ($^{31}P$-MRS) 6 h following initial administration of PEG–GO, revealed a 96 ± 2% reduction in the ATP/$P_i$ ratio and a 0.72 ± 0.10 unit decline in intracellular pH. A 3-fold sensitisation of 9L glioma cells *in vitro* to hydrogen peroxide could be achieved by a 24 h preincubation with buthionine sulphoximine (BSO). This study suggests that oxidation therapy, the use of an intratumoral ROS-generating enzyme system for the treatment of solid tumours, is a promising area which warrants further exploration.

Hydrogen peroxide is produced in mammalian cells during normal metabolism by different oxidases and from either spontaneous or superoxide dismutase (SOD)-catalysed superoxide anion dismutation. Hydrogen peroxide can readily cross cellular membranes, and the most sensitive cellular target structure for hydrogen peroxide is DNA, in which single-strand breaks occur (Hoffman & Meneghini, 1979; Bradley & Erickson, 1981; Imlay *et al.*, 1988). This genotoxicity has been proposed to be the result of transition metal-driven Haber–Weiss reactions leading to the generation of cytotoxic hydroxyl radicals (Filho & Meneghini, 1984). Over the past 40 years there have been many attempts to treat tumour-bearing patients or animals with preformed hydrogen peroxide (Hollcroft *et al.*, 1952; Makino & Tanaka, 1953; Turner, 1953; Green & Westrop, 1958; Sugiura, 1958; Krementz *et al.*, 1963; Mealey, 1965; Kaibara *et al.*, 1971). In these reports, hydrogen peroxide was injected directly into the solid tumour, the circulation or the peritoneal cavity of mice with malignant ascites. The results from these studies, however, were anecdotal. It was not until the early 1980s that investigators reported that hydrogen peroxide can exert a direct anti-tumour effect *in vivo* and thus prolong host survival (Nathan & Cohn, 1981). In that previous study, glucose oxidase (EC 1.1.3.4) was conjugated to carboxylated latex micropheres and was found to have an anti-tumour effect both in the peritoneal cavity and when injected into the tumour bed of subcutaneously implanted P388 cells at the time of cell implantation (Nathan & Cohn, 1981). The microspheres were at the tumour site and hydrogen peroxide generation was achieved through the following reaction:

$$\beta\text{-D-Glucose} + O_2 \xrightarrow{GO} \text{D-glucono-1,5-lactone} + H_2O_2$$

It is known that enzymes can be stabilised *in vivo* by attaching PEG units to the lysine residues. Enzyme modification in this manner increases *in vivo* half-life from 6 min to 30–40 h, reduces the antigenicity of the native protein and inhibits proteolysis (Pyatak *et al.*, 1980). To our knowledge, there have been no reports demonstrating growth inhibition of a solid tumour using hydrogen peroxide or a hydrogen peroxide-generating system. The unrealised potential of this treatment was the motivating factor for this present study, in which we describe the anti-tumour effect of PEG–GO on the growth of cultured 9L glioma cells and solid s.c. 9L tumours in rats. In addition, results using $^{31}P$-MRS to examine the effects of this therapeutic approach on tumour phosphate metabolites and pH *in vivo* are presented.

Correspondence: B.D. Ross.
Presented in part at the 12th Annual Meeting of the Society of Magnetic Resonance in Medicine, August 14–20, 1993, New York, NY.
Received 5 May 1994; and in revised form 22 July 1994.

### Materials and methods

### *Preparation of PEG–glucose oxidase (PEG–GO)*

The preparation of PEG–catalase, PEG–SOD and PEG–GO was accomplished using previously reported procedures for PEG–SOD (Pyatak *et al.*, 1980). In brief, PEG–GO was prepared by addition of 40 mg of protein (*Aspergillus niger*) in 10 ml of 0.1 M boric acid, pH 9.8. Activated PEG (1.0 g; $M_r$ 5,000, Sigma, St Louis, MO, USA) was added and the mixture was stirred for 1 h. Unattached PEG was removed by five sequential dialysis steps against equivalent volumes of phosphate-buffered saline (PBS) (calcium chloride, 0.9 mM; potassium chloride 2.7 mM; potassium dihydrogen phosphate 1.5 mM; magnesium chloride 0.3 mM; sodium chloride 137.0 mM; disodium hydrogen phosphate, 7.5 mM) using an Amicon (Beverly, MA, USA) ultrafiltration chamber (model 8050) equipped with an XM-50 membrane. The $M_r$ and activity of PEG–GO were determined by laser desorption linear time-of-flight mass spectrometry (Vestec Model VT 2000) and spectrophotometry (Michiels & Remacle, 1988) respectively. PEG–SOD and PEG–catalase were assayed for activity using standard spectrophotometric methods (Pyatak *et al.*, 1980; Michiels & Remacle, 1988).

### *Sulforhodamine B (SRB) cell toxicity assay*

Rat 9L glioma cells were grown until confluent as monolayers in 75 cm² sterile plastic flasks in modified Eagles minimum essential medium containing 10% fetal calf serum at 37°C in a humidified atmosphere containing 95% air and 5% carbon dioxide. Cells were harvested by trypsinisation, counted, diluted in serum-free medium, plated in 96-well culture plates at a density of 1,000 cells per well and allowed to grow for 24 h before treatment. Increasing concentrations of PEG–GO and hydrogen peroxide were subsequently added to the 9L cells in Krebs–Ringer bicarbonate buffer (KRB) sodium chloride 113.0 mM; sodium bicarbonate 29.0 mM; potassium chloride 4.6 mM; magnesium sulphate 0.5 mM; disodium hydrogen phosphate 0.6 mM; potassium hydrogen phosphate 0.3 mM; potassium bicarbonate 1.0 mM; calcium chloride 2.5 mM; glucose, 5.5 mM; pH = 7.4) for a 60 and 30 min exposure period respectively. The activity of PEG-GO was determined in KRB on the same day of cell exposures to ensure accurate dosing. An automated microculture assay using SRB (a protein-binding dye; Rubinstein *et al.*, 1990) was used to quantitate the toxicity of preformed and PEG-GO-generated hydrogen peroxide on cultured 9L glioma cells. Under the conditions used in this study, the optical absorbance was directly proportional to the number of 9L cells. Following hydrogen peroxide exposure, cells were gently rinsed three times and allowed to grow for 4 days. The cells were then fixed and stained, and the absorbance values were obtained using an AutoReader microtitre plate reader (Cayman Chemicals, Ann Arbor, MI, USA). Toxicity was assessed in terms of fractional cell survival relative to control.

Studies to evaluate the effects of reduced intracellular glutathione (GSH) levels on sensitivity of 9L tumour cells were done by preincubating cells for 24 h in the presence of 0.1, 0.5, 1, 2 and 5 mM buthionine sulphoximine (BSO) before exposure to increasing concentrations of hydrogen peroxide as described above. GSH levels were determined spectrophotometrically using a kit provided by Bioxytech (Marne, France).

### In vivo *evaluation of PEG-GO*

Specific pathogen-free male Fischer-344 rats were purchased from Charles River Breeding Laboratories (Wilmington, MA, USA). Rats weighed 275–300 g at the beginning of the experiment and were housed in the university laboratory animal medicine facility. All procedures were approved by the Institutional Animal Care and Use Committee. Subcutaneous gliomas were induced by injection of $1 \times 10^6$ 9L glioma cells in 0.2 ml of serum-free culture medium over the right thigh muscle under halothane anaesthesia. Calliper measurements of tumour dimensions were repeated every 2 days beginning on the eighth day post cell implantation. Tumour volumes were determined using the equation: Tumour volume = 0.5 (length × width$^2$). Rats with subcutaneous gliomas were treated 12 days after cell inoculation, at which time the mean tumour volume was approximately 0.7 cm$^3$. PEG-GO (50 or 200 U) contained in 50 µl of phosphate-buffered saline (PBS) was administered by an intratumoral injection. Tumours treated with 200 U of PEG-GO received a second identical injection 3 h later. Care was taken to distribute the PEG-GO as evenly as possible throughout the tumour mass. At the conclusion of the second 200 U PEG-GO injection, 10,000 U each of PEG-SOD and PEG-catalase were administered i.v. in order to protect the animals from systemic toxicity owing to the potential leakage of hydrogen peroxide and/or the PEG-GO from the tumour site into the vascular system. Control animals were treated identically except that the PEG-GO was denatured by heating at 90°C for 15 min prior to injection.

In order to determine the effects of this therapy on cellular energy state *in vivo*, $^{31}$P-MR spectra were recorded on several animals before and 6 and 24 h after intratumoral injection using a horizontal-bore 7 T Spectroscopy Imaging Systems magnetic resonance instrument. For MRS experiments, rats were anaesthetised by an i.p. injection of a mixture containing ketamine (80–100 mg kg$^{-1}$) and xylazine (13 mg kg$^{-1}$). $^{31}$P-MR spectra were recorded using a single-turn 9-mm diameter surface coil probe at 121.4 MHz using the following acquisition parameters: sweep width = ± 3,000 Hz; number of transients = 1,000; interpulse delay = 2.3 s; number of data points = 4096. $^{31}$P-MR spectra were apodised with a 30 Hz exponential line-broadening function prior to Fourier transformation. Quantification of ATP and inorganic phosphate ($P_i$) resonance areas was accomplished using a spectral deconvolution routine and pH was calculated from the chemical shift of $P_i$ relative to phosphocreatine (PCr). When the PCr resonance was absent, the water resonance from the proton spectra obtained during shimming procedures was used to calculate the position of PCr and hence pH as previously described (Madden *et al.*, 1991). Determination of pH values using this approach has been reported to be well within the expected accuracy of the NMR pH measurement of ± 0.05–0.1 pH units (Roberts *et al.*, 1981).

### *Statistical analysis*

Piecewise linear regression (Wasserman & Kutner, 1990) was used to model growth in tumour volume as a function of time, allowing for a different rate of growth in the treated group following treatment on day 12. The natural logarithm of tumour volume was used as the dependent variable because preliminary analysis indicated that the tumour volume increases in an approximately exponential manner with increasing time. The independent variables in the model were time in days since implantation and, for the treated group, time since treatment began. This variable was equal to zero both for the control group and for the treated group before treatment.

For $^{31}$P-MR data, all values are reported as means ± s.e.

### Results

### *Characterisation of PEG-GO*

The average $M_r$ of native GO and PEG-GO was determined to be 150 and 185 kDa respectively. Because the PEG used in this study had a $M_r$ of 5,000, an average of seven PEG moieties were attached to each PEG-GO molecule. Attachment of PEG reduced the enzymatic activity by 40% compared with native glucose oxidase as estimated from Lineweaver-Burk plots (data not shown).

### In vitro *cytotoxicity studies*

Survival studies of 9L glioma cells 4 days following exposure to increasing activities of PEG-GO for 60 min revealed that this enzyme system is cytotoxic to 9L glioma cells (Figure 1a). Cytotoxicity studies of 9L glioma cells following a 30 min exposure to preformed hydrogen peroxide revealed a dose-dependent toxicity with an $IC_{50}$ = 60µM ($n$ = 16, Figure 1b). In the presence of 0.1 mM BSO, an inhibitor of GSH synthesis, the $IC_{50}$ decreased to 22µM hydrogen peroxide, indicating an approximate 3-fold increased sensitivity to hydrogen peroxide (Figure 1b). Cell toxicity following a 24 h incubation with 0.5, 1, 2 and 5 mM BSO was identical to that observed for 0.1 mM BSO. Spectrophotometric determination of intracellular GSH levels under these conditions revealed a decreae from 42 to 20 nmol mg$^{-1}$ protein with 0.1 mM BSO. At BSO concentrations of 0.5 mM or higher, GSH was not detectable.

### In vivo *$^{31}$P-MRS and growth inhibitor studies*

Shown in Figure 2 are *in vivo* $^{31}$P-MR spectra of a subcutaneous 9L glioma acquired before PEG-GO injection (Figure 2a) and 6 h (Figure 2b) and 24 h (Figure 2c) following intratumoral injection of 50 U of PEG-GO. The spectrum obtained 6 h post intratumoral injection of 50 U PEG-GO shows a significant impairment of the energy state as indicated by the decrease in the PCr and ATP resonances and the increase in $P_i$. However, after 24 h, the energy state almost completely recovered and the rate of tumour growth remained unretarded. Shown in Figure 3 are representative *in vivo* $^{31}$P-MR spectra of subcutaneous 9L gliomas acquired before tumour treatment (Figure 3a) and 6 h following intratumoral injection of 2 × 200 U of denatured PEG-GO (Figure 3b) and active (2 × 200 U) PEG-GO (Figure 3c). Intratumoral injection of heat-denatured PEG-GO (Figure 3b) resulted in a 34 ± 17% ($n$ = 3) decline in tumour ATP/$P_i$ levels and a reduction in tumour pH by 0.10 ± 0.10 U ($n$ = 3). Administration of active PEG-GO resulted in the nearly complete loss of PCr and ATP and a reciprocal increase in $P_i$ (Figure 3c). For PEG-GO-treated animals, the

**Figure 1** Effect of preformed and PEG-GO-generated hydrogen peroxide on 9L glioma cell growth *in vitro*. **a**, 9L glioma cells were incubated for 1 h with increasing activity of PEG–GO and allowed to grow for 4 days ($n = 8$ s.e.). **b**, Control (O) and preincubated (□) (24 h with 0.1 mM BSO) 9L cells were incubated with increasing concentrations of preformed hydrogen peroxide for 30 min and allowed to grow for 4 days ($n = 16$ s.e.). Cytotoxicity was measured using the SRB assay and data are expressed as per cent of control cell growth.

**Figure 2** *In vivo* $^{31}$P-MR spectra of subcutaneous rat 9L gliomas obtained (**a**) before and (**b**) 6 h and (**c**) 24 h following a direct intratumoral injection of 50 U of PEG–GO. Resonance assignments are as follows: 1, phosphomonoesters; 2, $P_i$ 3, PCr; 4, γ-ATP and β-ADP; 5, α-ATP, α-ADP and NAD; 6, unidentified diphosphodiester, possibly UDP sugars; 7, β-ATP.

ATP/$P_i$ ratio decreased by 96 ± 2% ($n = 3$) and was accompanied by a 0.72 ± 0.10 ($n = 3$) decline in tumour pH. Shown in Figure 4 is a plot of the average 9L subcutaneous tumour volumes *vs* the number of days after cell implantation for the control (denatured PEG–GO) and PEG–GO-treated subcutaneous 9L gliomas. The difference in growth rates between control and treated groups beginning at day 12 is significant at $P < 0.001$.

Tumours treated with 2 × 200 U PEG-GO turned from pink to a dark-brown/black colour within approximately 4 h after initiation of the treatment, accompanied by a swelling of the tumour tissue. In contrast, no significant changes in the appearance and size of tumours injected with denatured PEG–GO were found. A swelling of the hind limb bearing the tumour mass was also seen following the treatment but disappeared 1–2 days later. Rats receiving more than approximately 100 U of PEG–GO, without infusion of the antioxidative enzymes (PEG–catalase and PEG–SOD), exhibited significant side-effects including swelling of the hind limb, lethargy and ultimately death.

### Discussion

The significant delay in tumour growth demonstrates the feasibility of oxidation therapy, which we define as the use of an intratumoral ROS-generating enzyme system for the treatment of solid tumours. Previous studies using direct injections of hydrogen peroxidase into solid tumours were unsuccessful (Green & Westrop, 1958). A study using glucose oxidase conjugated to latex microspheres did not report attempts at treating solid tumours but rather tumour cells in the peritoneal cavity and co-injection of tumour cells with the micropheres at the time of s.c. implantation (Nathan & Cohn, 1981). However, modification of GO and PEG resulted in only a 40% reduction in activity as compared with a 90% loss of enzyme activity when conjugated to latex microspheres (Nathan & Cohn, 1981). This reduction in activity, when combined with the increased volume taken up by the inert microsphere material, resulted in a net 4,000-fold decrease in total enzyme activity per volume as compared with the PEG–GO system. Although the latex microsphere system has the advantage of retaining the GO at the tumour site, the therapeutic effectiveness towards solid tumours (such as the 9L glioma) is probably limited since the injectable volume necessary to attain the dose used in this present study would exceed the tumour volume.

**Figure 3** Representative *in vivo* $^{31}P$-MR spectra of subcutaneous rat 9L gliomas obtained (**a**) before and 6 h following a direct intratumoral injection of 2 × 200 U of (**b**) heat-denatured PEG-GO and (**c**) active PEG-GO. Resonance assignments are as in Figure 2.

**Figure 4** Effect of intratumoral administration of PEG-glucose oxidase on the growth of 9L gliomas *in vivo*. 9L glioma cells (1 × $10^6$) were inoculated subcutaneously into the right flank of a Fischer 344 rat on day 0. PEG-glucose oxidase and heat-denatured PEG-glucose oxidase was administered intratumorally on day 12 (noted by upward pointing arrow) at two separate doses of 200 U 3 h apart. At the conclusion of the second PEG-glucose oxidase dose, 10,000 U each of PEG-catalase and PEG-SOD were administered i.v. to protect the rat from systemic oxidative stress. The difference in growth rates beginning at day 12 is significant at $P < 0.001$. Points, means ± s.e. ($n = 6$ control and $n = 5$ treated). O, heat denatured PEG-glucose oxidase; ●, 2 × 200 U PEG-glucose oxidase.

In this study, $^{31}P$-MRS was used to assess the *in vivo* metabolic effect of oxidation therapy since two distinct mechanisms by which hydrogen peroxide can deplete cellular ATP levels are known. The first mechanism involves activation of poly(ADP-ribose) polymerase following hydrogen peroxide-induced DNA strand breaks (Schraufstatter *et al.*, 1986). Activation of poly(ADP-ribose)polymerase consumes $NAD^+$, a process that is subsequently associated with a loss of ATP levels (Ueda & Hayaishi, 1985; Berger *et al.*, 1986; Bruchelt *et al.*, 1991). This second mechanism involves hydrogen peroxide inhibition of glycolysis by the inactivation of glyceraldehyde 3-phosphate dehydrogenase resulting in depletion of ATP levels (Brodie & Reed, 1987). Our results, obtained from *in vivo* $^{31}P$-MRS evaluation of treated gliomas, are consistent with the two mechanisms mentioned above in which ATP depletion is directly linked to oxidative stress. $^{31}P$-MR spectra of PEG-GO-treated 9L gliomas revealed a dramatic decline in both the ATP/$P_i$ ratio and tumour pH as compared with the control group at 6 h post treatment. The exact mechanism(s) for these changes could be one or a combination of the two aforementioned, however other possibilities, such as a decrease in tumour blood flow, cannot be ruled out. Whatever the exact mechanism(s) for cell death, it is important to note that metabolic changes preceded the inhibition of tumour growth, suggesting that $^{31}P$-MRS can provide a non-invasive means for following the progress of the treatment by monitoring ATP levels. This capability may be very useful during the actual treatment period by providing the feedback necessary to determine when the generation of ROS could be discontinued.

Optimisation of this therapeutic approach will require improvements in the delivery and control of the ROS-generating enzyme system and assessment of the pathological consequences. Glucose oxidase was chosen to demonstrate the concept of oxidation therapy because it has a relatively high specific activity, is commercially available and because the substrates necessary for hydrogen peroxide production, glucose and oxygen are abundant in tissue. However, this enzyme has a fundamental disadvantage in that its activity cannot be directly controlled, necessitating administration of large doses of antioxidative enzymes (or antioxidants) in order to protect the rat from systemic toxicity. This tissue may be exacerbated in the rat because blood catalase activity in this species is only 25% of that in the human (Lorincz *et al.*, 1948). This disparity between species is also reflected in the fact that human blood foams in contact with hydrogen peroxide, whereas this phenomenon does not occur when hydrogen peroxide is added to rat blood. Oxygenation of rat blood does not occur, but rather the blood turns dark-brown owing to the formation of methaemoglobin. Methaemoglobin formation was observed in the present studies in treated rats that were not protected by administration of PEG-catalase and PEG-SOD.

An alternative oxidase enzyme system in which the activity of the hydrogen peroxide generating system could be manipulated by substrate availability and/or direct chemical inhibition of the enzyme would provide a significant advantage over glucose oxidase. This would reduce excessive oxidative stress on surrounding normal tissues and blood. This capability would require that the substrate is not endogenous to mammalian tissue or present in low enough concentrations as not to induce significant formation of hydrogen peroxide. A specific example of an enzyme with these properties is D-amino acid oxidase (DAAO) (EC 1.4.3.3). This enzyme catalyses the following reaction and can be manipulated by substrate depletion or chemical inhibition:

$$\text{D-Amino acid} + H_2O + O_2 \xrightarrow{\text{DAAO}} \text{2-oxo acid} + NH_3 + H_2O_2$$

Therefore, the long-term development and optimisation of oxidation therapy will necessitate the use of an oxidase in which the activity can be manipulated.

Selectivity of oxidation therapy for gliomas will depend on the relative sensitivity of neurons, astrocytes and glioma cells to hydrogen peroxide-induced toxicity. We have obtained preliminary *in vitro* results showing the $IC_{50}$ of a 30 min exposure to hydrogen peroxide for rat primary neuronal and primary astrocytic cultures to be 98 and 585 $\mu$M respectively (O. Ben-Yoseph, P. Boxer & B.D. Ross, unpublished results), as compared with 60 $\mu$M for cultured 9L cells. We have also shown in this present study that sensitivity to hydrogen peroxide could be greatly increased (3-fold) when cultured 9L tumour cells were pretreated with BSO, (Griffith, 1982). BSO has an additional advantage of selectivity as it has been shown to reduce the GSH levels of intracranial gliomas without affecting normal brain GSH levels (Lippitz *et al.*, 1990). Thus, pretreatment of the host with BSO should provide an excellent method for the selective sensitisation of malignant brain tumours to oxidation therapy. Further methods for sensitising gliomas to oxidative stress include, for example, inhibition of tumour antioxidant defences or DNA repair mechanisms.

Future development of oxidation therapy will entail not only optimising the oxidase system and selective presensitisation of the tumour, but also improving methods of delivery to the tumour site. For treatment of malignant brain tumours, the disrupted blood–brain barrier should also allow an i.v.-administered PEG–oxidase system selective access to the glioma tissue. Intrathecal administration of the PEG–oxidase substrate could provide for activation of the enzyme predominantly within the tumour tissue. Progress of the therapy could be monitored using localised $^{31}$P-MRS *in vivo*. There are a multitude of additional variations on how oxidation therapy could be utilised for tumour treatment. Possible delivery methods include the use of liposomes, biodegradable polymers or attachment to monoclonal antibodies (Philpott *et al.*, 1980). Although the type of delivery vehicle will no doubt vary depending upon the tumour type and host organ targeted, our initial promising data provide evidence that oxidation therapy is a strong candidate for tumour treatment and warrants further evaluation.

We would like to thank Drs James A. Dykens and Peter B. Kingsley for helpful comments, Dr Judith Bromberg for statistical analysis and Mariliz Ortiz for technical assistance. 9L glioma cells were obtained from the Brain Tumour Research Center at the University of San Francisco. O. Ben-Yoseph is a Fellow of the American Brain Tumor Association. This study was supported in part by American Cancer society BE-149, NIH P20 NS31114-01, NIH R29 CA59009 and the University of Michigan Office of the Vice President for Research Grant No. 1954.

### References

BERGER, N.A. (1986). Cancer chemotherapy: new strategies for success. *J. Clin. Invest.*, **78**, 1131–1135.

BRADLEY, M.O. & ERICKSON, L.C. (1981). Comparison of the effects of hydrogen peroxide and X-ray irradiation on toxicity, mutation, and DNA damage/repair in mammalian cells (V-79). *Biochim. Biophys. Acta.*, **654**, 135–141.

BRODIE, A.E. & REED, D.J. (1987). Reversible oxidation of glyceraldehyde 3-phosphate dehydrogenase thiols in human lung carcinoma cells by hydrogen peroxide. *Biochem. Biophys. Res. Commun.*, **148**, 120–125.

BRUCHELT, G., SCHRAUFSTATTER, I.U., NIETHAMMER, D. & COCHRANE, C.G. (1991). Ascorbic acid enhances the effects of 6-hydroxydopamine and $H_2O_2$ on iron-dependent DNA strand breaks and related processes in the neuroblastoma cell line SK-N-SH. *Cancer Res.*, **51**, 6066–6072.

FILHO, A.C.M. & MENEGHINI, R. (1984). *In vivo* formation of single-strand breaks in DNA by hydrogen peroxide is mediated by the Haber–Weiss reaction. *Biochim. Biophys. Acta.*, **781**, 56–63.

GREEN, N.H. & WESTROP, J.W. (1958). Hydrogen peroxide and tumour therapy. *Nature*, **181**, 128–129.

GRIFFITH, O.W. (1982). Mechanism of action, metabolism and toxicity of buthionine sulfoximine and its higher homologs: potent inhibitors of glutathione synthesis. *J. Biol. Chem.*, **257**, 13704–13709.

HOFFMANN, ME. & MENEGHINI, R. (1979). Action of hydrogen peroxide on human fibroblast in culture. *Photochem. Photobiol.*, **30**, 151–155.

HOLLCROFT, J.W., LORENZ, E. & MATTHEWS, M. (1952). Factors modifying the effect of X-irradiation on regression of a transplanted lymphosarcoma. *J. Natl Cancer Inst.*, **12**, 751–763.

IMLAY, J.A., CHIN, S.M. & LINN, S. (1988). Toxic DNA damage by hydrogen peroxide through the Fenton reaction *in vivo* and *in vitro*. *Science*, **240**, 640–642.

KAIBARA, N.T., IKEDA, T., HATTORI, T. & INOKUCHI, K. (1971). Experimental studies on enhancing the therapeutic effect of mitomycin-C with hydrogen peroxide. *Jpn J. Exp. Med.*, **41**, 323–329.

KREMENTZ, E.T., YOUNGBLOOD, J.W. & HEALY, W.R. (1963). The effect of peroxides, peroxidase inhibitors, and alkylating agents on ascites tumours in mice (abstract). *Proc. Am. Assoc. Cancer Res.*, **4**, 36.

LORINCZ, A.L., JACOBY, J.J. & LIVINGSTONE, H.M. (1948). Studies on the parenteral administration of hydrogen peroxide. *Anesthesiology*, **9**, 162–174.

LIPPITZ, B.E., HALPERIN, E.C., GRIFFITH, O.W., COLVIN, O.M., HONORE, G., OSTERAG, C.B., BIGNER, D.D. & FRIEDMAN, H.S. (1990). L-Buthionine-sulfoximine-mediated radiosensitization in experimental interstitial radiotherapy of intracerebral D-54 MG glioma xenografts in athymic mice. *Neurosurgery*, **26**, 255–260.

MADDEN, A., LEACH, M.O., COLLINS, D.J. & PAYNE, G.S. (1991). The water resonance as an alternative pH reference: relevance to *in vivo* $^{31}$P-NMR localized spectroscopy studies. *Magn. Reson. Med.*, **19**, 416–421.

MAKINO, S. & TANAKA, T. (1953). The cytological effects of chemicals on ascites sarcomas. II. Selective damage to tumour cells by $CaCl_2$ $AlCl_3$ and $H_2O_2$. *Gann.*, **44**, 39–46.

MEALEY, J. (1965). Regional infusion of vinblastine and hydrogen peroxide in tumour-bearing rats. *Cancer Res.*, **25**, 1839–1843.

MICHIELS, C. & REMACLE, J. (1988). Use of the inhibition of enzymatic antioxidant systems in order to evaluate their physiological importance. *Eur. J. Biochem.*, **177**, 435–441.

NATHAN, C.F. & COHN, Z.A. (1981). Antitumour effects of hydrogen peroxide *in vivo*. *J. Exp. Med.*, **154**, 1539–1553.

PHILPOTT, G.W., KULCZYCKI, Jr, A., GRASS, E.H. & PARKER, C.W. (1980). Selective binding and cytotoxicity of rat basophilic leukemia cells (RBL-1) with immunoglobulin E-biotin and avidin-glucose oxidase conjugates. *J. Immunol.*, **125**, 1201–1209.

PYATAK, P.S., ABUCHOWSKI, A. & DAVIS, F.F. (1980). Preparation of a polyethylene glycol: superoxide dismutase adduct, and an examination of its blood circulating life and anti-inflammatory activity. *Res. Commun. Chem. Pathol. Pharmacol.*, **29**, 113–127.

ROBERTS, J.K.M., WADE-JARDETZKY, N. & JARDETSKY, O. (1981). Intracellular pH measurements by $^{31}$P nuclear magnetic resonance. Influence of factors other than pH on $^{31}$P chemical shifts. *Biochemistry*, **20**, 5389–5394.

RUBINSTEIN, L.V., SHOEMAKER, R.H., PAULL, K.D., SIMON, R.M., TOSINI, S., SKEHAN, P., SCUDIERO, D.A., MONKS, A. & BOYD, M.R. (1990). Comparison of *in vitro* anticancer-drug-screening data generated with a tetrazolium assay versus a protein assay against a diverse panel of human tumour cell lines. *J. Natl Cancer Inst.*, **82**, 1113–1118.

SCHRAUFSTATTER, I.U., HYSLOP, P.A., HINSHAW, D.B., SPRAGG, R.G., SKLAR, L.A. & COCHRANE, C.G. (1986). Hydrogen peroxide-induced injury of cells and its prevention by inhibitors of poly(ADP-ribose) polymerase. *Proc. Natl Acad. Sci. USA*, **83**, 4908–4912.

SUGUARA, K. (1958). Effect of hydrogen peroxide on transplanted rat and mouse tumours. *Nature*, **182**, 1310–1311.

TURNER, F.C. (1953). Studies with various experimental tumours. *Cancer Res.*, **13** (Suppl. 1), 81.

UEDA, K. & HAYAISHI, O. (1985). ADP-ribosylation. *Annu. Rev. Biochem.*, **54**, 73–100.

WASSERMAN, N.J. & KUTNER, M.H. (1990). *Applied Linear Statistical Models*, 3rd Edn, Richard D. Irwin: Homewood, IL.